# Mastocytosis and Mast Cell Activation Disorders: Clearing the Air

**DOI:** 10.3390/ijms222011270

**Published:** 2021-10-19

**Authors:** Clayton Webster Jackson, Cristina Marie Pratt, Chase Preston Rupprecht, Debendra Pattanaik, Guha Krishnaswamy

**Affiliations:** 1 Department of Medicine, Wake Forest School of Medicine, Winston-Salem, NC 27101, USA; cwjackso@wakehealth.edu (C.W.J.); cpratt@wakehealth.edu (C.M.P.); 2 The Department of Medicine, Rowan School of Osteopathic Medicine, Stratford, NJ 08084, USA; ruppre86@rowan.edu; 3 The Division of Allergy and Immunology, UT Memphis College of Medicine, Memphis, TN 38103, USA; dpattana@uthsc.edu; 4 The Bill Hefner VA Medical Center, The Division of Allergy and Immunology, Salisbury, NC 28144, USA

**Keywords:** anaphylactic shock, hypotension, allergic reaction, angioedema, mastocytosis, mast cell, tryptase, histamine

## Abstract

Mast cells are derived from hematopoietic stem cell precursors and are essential to the genesis and manifestations of the allergic response. Activation of these cells by allergens leads to degranulation and elaboration of inflammatory mediators, responsible for regulating the acute dramatic inflammatory response seen. Mast cells have also been incriminated in such diverse disorders as malignancy, arthritis, coronary artery disease, and osteoporosis. There has been a recent explosion in our understanding of the mast cell and the associated clinical conditions that affect this cell type. Some mast cell disorders are associated with specific genetic mutations (such as the D816V gain-of-function mutation) with resultant clonal disease. Such disorders include cutaneous mastocytosis, systemic mastocytosis (SM), its variants (indolent/ISM, smoldering/SSM, aggressive systemic mastocytosis/ASM) and clonal (or monoclonal) mast cell activation disorders or syndromes (CMCAS/MMAS). Besides clonal mast cell activations disorders/CMCAS (also referred to as monoclonal mast cell activation syndromes/MMAS), mast cell activation can also occur secondary to allergic, inflammatory, or paraneoplastic disease. Some disorders are idiopathic as their molecular pathogenesis and evolution are unclear. A genetic disorder, referred to as hereditary alpha-tryptasemia (HαT) has also been described recently. This condition has been shown to be associated with increased severity of allergic and anaphylactic reactions and may interact variably with primary and secondary mast cell disease, resulting in complex combined disorders. The role of this review is to clarify the classification of mast cell disorders, point to molecular aspects of mast cell signaling, elucidate underlying genetic defects, and provide approaches to targeted therapies that may benefit such patients.

## 1. Introduction

Since the discovery of mast cells (MCs) in the nineteenth century, our knowledge about these fascinating and multifunctional cells has grown exponentially [1,2,3,4,5]. Over the last few decades, disease associated with mast cell function and biology has been described. Mast cells orchestrate typical allergic conditions wherein activation of these cells by allergens (such as food, Hymenoptera venom, latex, antibiotics, and pollen) leads to degranulation and elaboration of inflammatory mediators, responsible for regulating the acute dramatic inflammatory response seen [6,7,8]. Mast cells modulate angiogenesis, tissue inflammation and repair, innate and adaptive immune responses, immune tolerance, and host defense [9]. As a consequence, mast cells have also been incriminated in many diverse disorders such as Crohn’s disease, malignancy, autoimmunity (including Guillain–Barré syndrome, Sjögren syndrome, vasculitis, and inflammatory arthritis), multiple sclerosis, coronary artery disease, arterial aneurysms, and osteoporosis [1,3,9,10,11]. Expansion of mast cell populations also occurs in chronic infections (parasitic infestation, tuberculosis, and syphilis), melanomas, chronic renal and hepatic disease, and in scleroderma [12].

Some mast cell disorders are associated with specific genetic mutations with resultant clonal disease (Table 1). Such disorders include cutaneous mastocytosis, systemic mastocytosis (SM), its variants (indolent/ISM, smoldering/SSM, aggressive/ASM), and some forms of the recently described mast cell activation disorders or syndromes (MCAD or MCAS) [13,14,15,16,17,18,19,20,21,22,23,24]. The latter include clonal mast cell activations disorders/CMCAS (also referred to as monoclonal mast cell activation syndromes/MMAS), mast cell activation secondary to allergic, inflammatory, or paraneoplastic disease, and idiopathic disorders [16,22,25,26,27,28]. These conditions have been better understood by the medical community in the last few decades due to advances in cellular biology and molecular techniques, leading to improved attempts at classification and prognostication [15,29,30,31]. A commonly observed gain of function mutation seen (KIT D816V) is a somatic missense A to T mutation and involves substitution of aspartic acid (D) to valine (V) at amino acid 816 in exon 17 of the KIT gene (a proto-oncogene also referred to as c-KIT), which encodes the KIT protein (CD117 or stem cell factor receptor/SCFR), a receptor tyrosine kinase. This transmembrane receptor binds to stem cell factor (SCF), initiating a signaling cascade in mast cells, regulating their growth, development, migration, and proliferation. In some situations, clonality is also determined by aberrant cellular expression of CD25 (alpha-chain of the IL-2 receptor) or CD2 (lymphocyte function antigen-2 or LFA-2) on neoplastic mast cells. Besides the above cluster of conditions, hereditary alpha-tryptasemia (HαT), described recently, has been shown to be associated with increased severity of allergic and anaphylactic reactions.

This article attempts a translation clinical-molecular review of an area that has rapidly evolved but has been compartmentalized in the literature, making it confusing. We have combined information on mastocytosis, mast cell sarcoma, mast cell activation disorders, hereditary alpha-tryptasemia, and idiopathic allergic/mast cell disorders in one place, with updated criteria for diagnosis and an overview of management. The role of this review, therefore, is to clarify the classification of mast cell disorders, point to molecular aspects of development, and provide evidence for broad targeted therapies that may benefit such patients. This is not meant to be an exhaustive review of mast cell biology or mast cell-related pathology.

## 2. Brief Overview of Mast Cell Biology

Human mast cells, first described by Paul Ehrlich, are multifunctional immune cells of myeloid lineage, intimately involved in allergic reactions [1,2,3,5,32,33]. Mast cells originate from CD34^+^/CD117^+^/CD13^+^ multipotent, hematopoietic progenitors [4,5]. They migrate to peripheral tissues and undergo differentiation and maturation under the influence of growth factors, including stem cell factor (SCF), where interactions with other cell types, including eosinophils and T cells, occur [2,3,4,5,34,35]. SCF is a mast cell chemoattractant that also influences the proliferation, differentiation, maturation, adhesion, and survival of mast cells. SCF binds to its receptor, KIT, a transmembrane tyrosine kinase-linked receptor and signal [36,37,38,39]. Somatic mutations in c-KIT that code for the KIT receptor (the most common of these being the D816V mutation) have been linked to the development of systemic mastocytosis, a clonal hematological disorder [40].

Under the influence of local tissue-derived maturation factors, mast cell differentiation results in the development of well-recognized phenotypes: mucosal mast cells (mainly tryptase-expressing or MCT cells, located predominantly in the mucosa) and alveoli and connective tissue mast cells (expressing tryptase, chymase, and carboxypeptidase A3 or MC-TC, located in submucosal and connective tissue sites such as skin) [2,3,4,5]. Atiakshin and co-workers have demonstrated the colocalization of tryptase and chymase in the same mast cell granules using sophisticated immunohistochemical techniques [41]. In Th2-mediated diseases, such as atopic asthma and nasal polyposis, intraepithelial MC-T cells may undergo structural changes. The unique co-expression of tryptase and carboxypeptidase A3 has been demonstrated by MC-T cells under these circumstances [42]. Dvorak has described finer aspects of mast cell ultrastructure, such as expression of scroll-containing granules, crystal-containing granules, particle-containing granules, particle-filled granules, or mixed types observed under electron microscopy [43].

Mast cells undergo maturation in target tissues under the influence of interleukin (IL)-3, IL-4, IL-9, IL-33, CXCL2, SCF, nerve growth factor (NGF), and transforming growth factor beta (TGF-β1) [4,5]. Autocrine expression of SCF by mast cells is postulated to represent mast cells regulating their own growth and survival [5].

**Figure 1:**
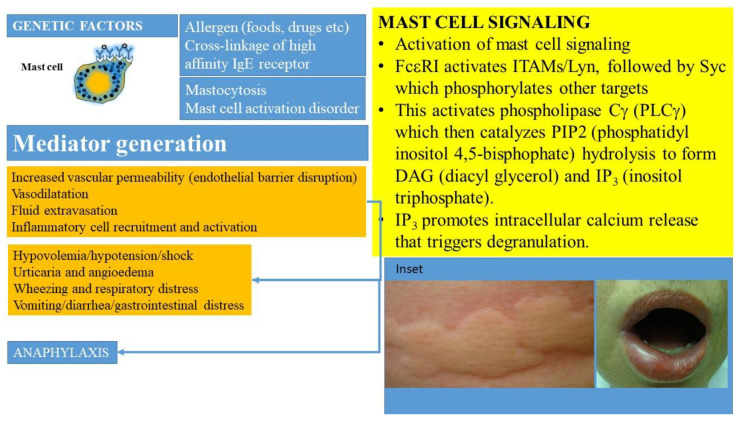
Activation of MCs occurs when allergen specific IgE is bound by the allergen and interacts with the high affinity receptor for immunoglobulin (IgE), referred to as FcεRI, carried on their surfaces in the presence of mutations and other molecular-genetic defects. This culminates in the release of preformed and newly synthesized mediators from mast cells and basophils that sets off a sequence of inflammatory events manifesting clinically as anaphylaxis and leading to shock. MC-derived cytokines, histamine, leukotrienes, prostanoids, and PAF regulate vascular instability and barrier dysfunction of endothelial cells and contribute to edema formation. Hypovolemia, angioedema, hypotension, and cardiorespiratory failure ensue. Inset shows examples of urticaria and angioedema.

Mast cell activation can occur following many triggers and via a plethora of cell surface receptors, including receptors for IgE, IgG, complement, cytokines, growth factors, stem cell factor, and neuropeptides (beyond the scope of this review) [4,11,33]. The prototypic IgE-mediated activation (Figure 1) occurs when allergen specific IgE is bound by a multivalent allergen and interacts with the high affinity receptor for immunoglobulin E (FcεR1) carried on their surfaces [44,45]. The high affinity receptor for IgE has a heterotetrametric structure, comprising an α-chain (binds IgE), a β-chain (plays a key role in signal transduction), and two γ-chains (that initiate signaling post-activation) [45]. This process activates a downstream signaling cascade (summarized in Figure 1) involving immunoreceptor tyrosine-based activation motifs (ITAMS) associated with the β- and γ-chains. The activation of tyrosine protein kinase Lyn leads to phosphorylation of Syc. Subsequent activation of phospholipase Cγ (PLCγ) catalyzes PIP2 (phosphatidyl inositol 4,5-bisphophate) hydrolysis to form DAG (diacyl glycerol) and IP_3_ (inositol triphosphate). IP_3_ promotes intracellular calcium release that triggers degranulation. This culminates in the release of preformed and neosynthesized mediators from mast cells and basophils that set off a sequence of inflammatory events manifesting clinically as anaphylaxis and leading to distributive shock (Figure 1). Genetic factors such as polymorphisms in the angiotensin converting enzyme, HαT, platelet activating factor defects (such as deficiencies of platelet activating factor acetyl hydrolase), cytokine gene polymorphisms, and the KARS mutation may modulate the severity of such reactions.

The degranulation of mast cells leads to the release of preformed mediators: histamine, serotonin, heparin, chondroitin sulfate, tryptase, chymase, carboxypeptidase, cathepsin G, prostaglandin D2 (PGD2), interleukin-4 (IL-4), and TNF-α [4,5,17,46,47]. Of these, the roles of tryptase, heparin, and histamine have been the most studied, though researchers have also evaluated the production of chymase and other mediators. This degranulation is followed by the release of de novo synthesized mediators such as lipid mediators (cysteinyl leukotrienes LTC 4, D4, and E4), platelet activating factor (PAF), cytokines (TNF-α, GM-CSF, IL-1, IL-3, IL-5, IL-4/IL-13, IL-6, IL-8, and IL-10), chemokines (such as IL-8, CCL-2, CC-3, CCL-5, and CXCL-8), growth factors (such as transforming growth factor beta 1 (TGF-β1), stem cell factor (SCF), fibroblast growth factor (FGF), nerve growth factor (NGF), platelet derived growth factor (PDGF), vascular endothelial growth factor (VEGF)), and interferons (interferon β and γ) [4,5,35].

Atiakshin and others have evaluated the secretome involving chymase, tryptase, and carboxypeptidase, but detailed descriptions of these are beyond the scope of this review and the reader is referred to other publications [4,18,48,49,50]. Tryptase, a neural serine protease that exists as two isoforms, alpha and beta, encoded by separate genes (TPSB2 and TPSAB1), is stored in secretory granules, often in association with other mediators [51]. Tryptase is released from secretory granules as inactive proenzymes (alpha- and beta-protryptase) following mast cell activation by IgE-dependent and independent processes. Beta-tryptases, unlike the alpha isoform, are released as a tetramer that is bound to heparin and chondroitin sulfate. This is the dominant form measured in commercial assays for total tryptase. Tryptase release is coordinated with the development of allergic and inflammatory responses, the latter being associated with tissue remodeling [48]. Tryptase has many biological effects, including the inactivation of substrates (fibrinogen, fibronectin, collagens, and lipoproteins), regulation of mesenchymal cell proliferation and survival, upregulation of adhesion molecules, and induction of growth factors and cytokines [51].

The roles of mediators in the clinical manifestations of mast cell disorders have been extensively reviewed by Valent, Butterfield, and others [8,52]. MC-derived cytokines, histamine, leukotrienes, prostanoids, and PAF, regulate vascular instability, the barrier dysfunction of endothelial cells, and contribute to edema formation, hypovolemia, and shock (Figure 1) [5,8]. Histamine, specifically, can contribute to many classic manifestations associated with acute allergic reactions, including those seen in mast cell activation syndromes, such as headache, nausea, pruritus, flushing, gastric hypersecretion, nasal congestion, and wheezing. Histamine has been shown to bind to four receptors: H1 (responsible for airway and mucosal inflammation, attention, sleep–wake cycle regulation, and food or water intake), H2 (involved in the relaxation of the airway and blood vessel smooth muscle and in gastric acid secretion), H3 (involved in neuroregulation and mediator release modifying cognition, sleep–wake cycle regulation, and inflammation), and H4 (involved in modulatory effects on inflammatory responses) [8]. Histamine, kinins, leukotrienes, and PAF may be responsible for the opening of endothelial gap junctions resulting in angioedema, pulmonary edema, and gastrointestinal symptoms (such as crampy abdominal pain and diarrhea related to mucosal swelling). Histamine, PGD_2_, leukotrienes, PAF, and prostanoids are responsible for wheezing and mucus hypersecretion. Cytokines, chemokines, leukotrienes, and histamine may lead to neurological symptoms including headaches, fatigue, sense of impending doom, and confusion [52]. Studies suggest that the activation of the contact system and the secretion of the plasminogen activator and heparin may lead to coagulation abnormalities and bleeding.

Mast cells also express other receptors on their surfaces, which have multiple functions that integrate complex inflammatory responses with cell survival and growth. Some pivotal cell surface receptors include those for cytokines (IL-33, IL-12, IL-10, interferon gamma/IFNγ), chemokines (CCR1-CCR7, CXCR1-6), complement (C3a/C5a, CR1-5), Toll-like receptors (TLR 1-6), histamine, prostaglandin, stem cell factor, and IgE [4]. Descriptions of these receptors and their functions are beyond the scope of this review [4,33,45,53].

## 3. Stem Cell Factor and Its Receptor

**Figure 2:**
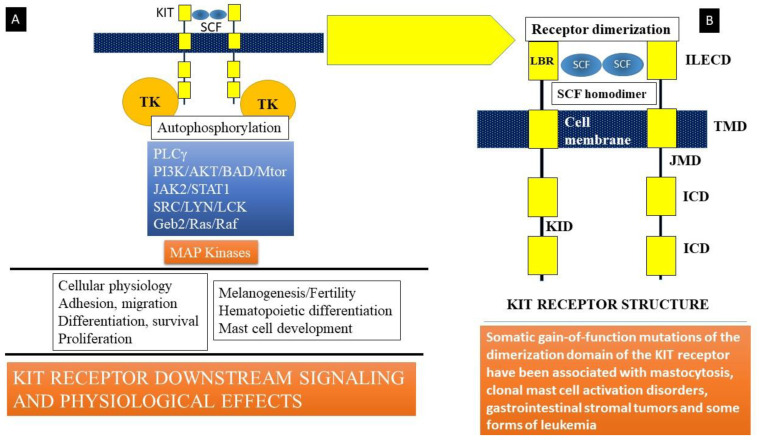
(**A**) Stem cell factor (SCF) is a hematopoietic growth factor that binds to its receptor, KIT, a transmembrane tyrosine kinase-linked receptor on mast cells. Signaling downstream involves activation of key kinases (Phospholipase Cγ (PLCγ), protein kinase C (PKC), and linked signaling proteins: Janus kinase 2 (a non-receptor tyrosine kinase, JAK2), signal transducer and activator of transcription 1 (STAT1), and other signal transduction mediators). These lead to action of mitogen-activated protein kinases (MAP kinase). Somatic mutations in c-KIT that code for the KIT receptor have been linked to the development of systemic mastocytosis, a clonal hematological disorder. The most common of these mutations is the D816V mutation that leads to enhanced survival and proliferation of mast cells, a feature of clonal mast cell disorders including mastocytosis and mast cell activation disorders. (**B**) KIT is the cellular counterpart of the v-KIT oncogene derived from a feline leukemia virus. It is encoded in the W or c-KIT locus on human chromosome 4q11-q12. KIT is composed of an immunoglobulin-like extracellular domain (ILECD), that is involved in ligand binding (LBR), an anchoring transmembrane domain (TMD), a juxta-membranous domain (JMD), and intracellular domains (ICD). KID = kinase insert domain. Binding of stem cell factor to KIT results in receptor dimerization and activation of protein kinase activity.

Stem cell factor (SCF) is a hematopoietic growth factor bound to its receptor, KIT, a transmembrane tyrosine kinase-linked receptor on mast cells [4,54]. KIT is the cellular counterpart of the v-KIT oncogene derived from the feline leukemia virion. It is encoded in the W or c-KIT locus on human chromosome 4q11-q12. KIT (Figure 2) is composed of an immunoglobulin-like extracellular domain (ILECD with five components) that is involved in ligand (SCF) binding, an anchoring transmembrane domain (TMD), a juxta-membranous domain (JMD), an intracellular domain (ICD), and a kinase insert domain (KID). The binding of stem cell factor to KIT results in receptor dimerization and the activation of protein kinase activity. Signaling downstream involves the activation of key kinases, such as phospholipase Cγ (PLCγ), protein kinase C (PKC), and linked signaling proteins such as Janus kinase 2 (a non-receptor tyrosine kinase or JAK2), signal transducer and activator of transcription 1 (STAT1), and others. These mechanisms lead to the action of mitogen-activated protein kinases (MAP kinases), resulting in multiple cellular effects such as adhesion, migration, differentiation, survival, and proliferation of mast cells (Figure 2). Somatic mutations in c-KIT that code for the KIT receptor have been linked to the development of systemic mastocytosis, a clonal hematological disorder. The most common of these mutations is the D816V mutation that leads to enhanced survival and proliferation of mast cells, a feature of clonal mast cell disorders, including mastocytosis and mast cell activation disorders.

The following review discusses the complex plethora of mast cell disorders, including clonal mast cell disorders, mast cell activation disorders, hereditary alpha-tryptasemia, and idiopathic disease. This review also provides an approach to their classification, diagnosis, presentation, management, and prognosis.

## 4. Materials and Methods

A MeSH search for the word “mastocytosis” yielded 7732 results, “systemic mastocytosis” yielded 2525 results, “cutaneous mastocytosis” yielded 2278 results, and “mast cell activation/syndromes” yielded 7789 results on PubMed. MeSH searches were also conducted for mast cell activation syndrome(s), mast cell activation disorders, tryptase, hereditary alpha-tryptasemia, and mast cell-related anaphylaxis. We chose papers published in the last two decades with a focus on published guidelines, systematic reviews, and reviews of pathophysiology. For mechanisms, when relevant, both animal and human studies were included. Additionally, searches for the therapy or management of mast cell disorders were used to present current attempts at precision medicine and targeted therapies for mast cell disorders.

## 5. Classification

Table 1 classifies the disorders that have been described to date involving mast cells. Some of these disorders are clonal in nature and are associated with either mutations of c-KIT (commonly D816V) and/or expression of CD25 on mast cell surfaces, detected by either flow cytometry or immunocytochemistry. Some disorders are hereditary, but not necessarily clonal (such as the autosomal dominant disorder hereditary alpha tryptasemia, or HαT syndrome, a condition that may complicate clonal or non-clonal mast cell disorders), and others are neither (such as allergic and autoimmune disorders, neoplasia, paraneoplastic syndromes, and idiopathic mast cell activation). We will discuss cutaneous mastocytosis (CM), systemic mastocytosis (SM) and its variants, mast cell activating syndrome (MCAS) with its various components, idiopathic anaphylaxis (IA), the unique conundrum of Hymenoptera allergy, the unusual disorder referred to as bone marrow mastocytosis (BMMC), and hereditary alpha-tryptasemia syndrome (HαT).

## 6. Mastocytosis 

**Figure 3:**
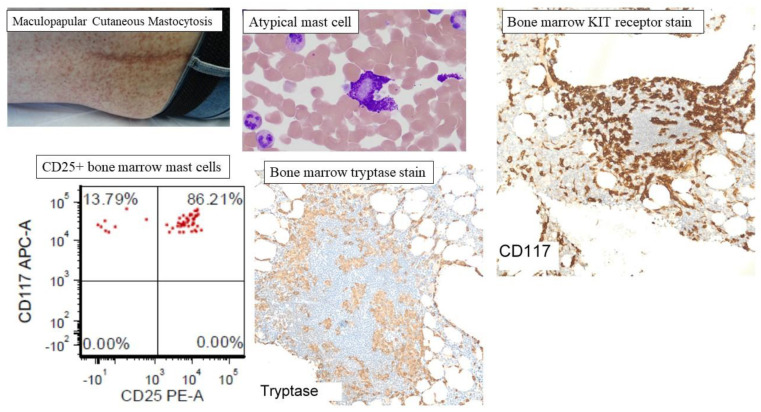
Representative clinicopathological features of mastocytosis. Cutaneous mastocytosis is subcategorized as maculopapular cutaneous mastocytosis (MPCM), also known as urticaria pigmentosa or diffuse cutaneous mastocytosis (DCM), and mastocytoma of the skin. MPCM is the most common presentation of CM with hyperpigmented nodules, papules, or macules distributed randomly, and appearing most commonly on the trunk. The figure shows representative atypical mast cells, mast cell aggregates in the bone marrow (CD117 and tryptase staining), and CD25+ atypical mast cells on flow cytometry of bone marrow aspirate.

Mastocytosis (Box 1 and Box 2) encompasses a heterogenous collection of disorders characterized by proliferation and activation of atypical mast cells with variable organ system involvement [29]. Traditionally, mastocytosis has been broadly categorized as cutaneous mastocytosis (CM) or systemic mastocytosis (SM) (Figure 3) [55]. Mast cell infiltration involving at least one extracutaneous organ system, of which the bone marrow is frequently affected, is the characteristic feature which distinguishes SM from CM [29,55,56]. The clinical presentation is diverse, with disease burden ranging from isolated skin involvement in CM, to more advanced subtypes with evidence of infiltrative organ dysfunction [29,55].

Box 1 **Criteria for Diagnosis of Systemic Mastocytosis.**

Systemic mastocytosis (SM)/Indolent Systemic Mastocytosis (ISM)

Diagnosis is confirmed if patient expresses one major criterion and one minor criterion or expresses three minor criteria

 

Major criteria:

Multifocal dense infiltrates of mast cells (>15 cells/aggregate) in bone marrow or extramedullary, extracutaneous tissue

 

Minor criteria:

1. >25% of infiltrating mast cells are atypical or spindle-shaped
2. KIT mutation in bone marrow or peripheral blood or extracutaneous tissue
  - Other rarer mutations may be observed
  - These include TET2, SRSF2, ASXL1, RUNX1, CBL, JAK2
3. Mast cells express clonal markers such as CD25 or CD2 by immunocytochemistry or flow cytometry (besides CD117 or tryptase)
4. Serum tryptase is >20 ng/mL (unless there is an underlying myeloid neoplasm)

Box 2 **Criteria for other Variants of Mastocytosis and Leukemia.**

Smoldering Systemic Mastocytosis (SSM)

1. Meets all criteria for systemic mastocytosis
2. Additional 2 or more type B symptoms are present
  - >30% of cells in bone marrow or extracutaneous tissue are mast cells and tryptase levels are >200 ng/mL
  - Dysplasia or myeloproliferation of non-mast cell lineage cells but not meeting criteria for hematological neoplasm
  - Organomegaly (hepatomegaly, splenomegaly, lymphadenopathy) in the absence of functional organ impairment

SystemicMastocytosis with Associated Hematological Neoplasm (SM-AHN)

1. Meets criteria for systemic mastocytosis
2. Meets criteria for associated hematological neoplasm

Aggressive Systemic Mastocytosis (SM)

1. Meets criteria for systemic mastocytosis
2. Additional 1 or >  C type findings but no evidence for mast cell leukemia
  - Bone marrow dysfunction due to mast cell infiltration with associated cytopenia (neutropenia, anemia, thrombocytopenia)
  - Hepatomegaly with abnormal liver functions, portal hypertension or ascites
  - Osteolytic lesions with associated pathological fractures not related to osteoporosis
  - Palpable splenomegaly with hypersplenism and cytopenia
  - Gastrointestinal infiltration by mast cells with malabsorption

Mast Cell Leukemia

1. Meets criteria for systemic mastocytosis
2. Bone marrow is densely infiltrated by atypical or immature mast cells
  - Bone marrow aspirate shows mast cells >30%
  - Mast cells may represent >10% of circulating white cells in blood except in aleukemic variant

Mast Cell Sarcoma

1. Does not meet criteria for mastocytosis
2. Highly malignant atypical mast cells with destructive and metastatic potential
3. Mast cell activation symptoms are rare (<20%)
4. Minority of patients express the canonical D816V mutation

## 7. Clonal Disorders: Cutaneous Mastocytosis

Cutaneous mastocytosis (CM) occurs most commonly in childhood with a good prognosis [57]. Most cases occur before the age of two and often resolve spontaneously, unlike the adult counterparts which are usually associated with bone marrow disease [24,58]. Based on macroscopic features, distribution, and the timing of lesions, CM is subcategorized as maculopapular cutaneous mastocytosis (MPCM), also known as urticaria pigmentosa, diffuse cutaneous mastocytosis (DCM), and mastocytoma of the skin [57]. MPCM is the most common presentation of CM with hyperpigmented nodules, papules, or macules distributed randomly, appearing most commonly on the trunk (Figure 3). Mastocytoma is described as one or two raised nodules or plaques. DCM is the least common cutaneous variant, although the most severe subtype, manifesting with diffusely thickened, erythematous skin markings with vesicles and edema due to overwhelming mast cell infiltration affecting the skin [59]. Darier’s sign is a physical examination feature of mastocytosis described as the formation of a wheal in response to rubbing, scratching, or stroking of the skin. Darier’s sign is present in most cases of mastocytosis affecting the skin, although the absence of Darier’s sign does not rule out a diagnosis [60]. When occurring in childhood, the demonstration of cutaneous mast cell involvement is enough to support a diagnosis of CM without further evaluation; however, mastocytosis of the skin developing in adulthood is often an extension of SM, usually indolent SM, and warrants further diagnostic evaluation with a bone marrow biopsy [23,24,55,57]. 

## 8. Clonal Disorders: Systemic Mastocytosis and Advanced Systemic Mastocytosis 

The major criterion (Box 1) for diagnosis of SM is the presence of multifocal dense infiltrates of mast cells (MC) which is defined as ≥15 MCs/aggregate in bone marrow (Figure 3) or other extracutaneous tissue biopsy. Minor criteria include the proportion of atypical (Figure 3) or spindle-shaped MCs exceeding 25% of all MCs, identification of the oncogenic *KIT* D816V mutation, MC expression of CD2 and/or CD25 (Figure 3), and baseline serum tryptase level > 20 ng/mL. The presence of one major and one minor criteria, or the presence of three minor criteria, is needed to support a diagnosis of SM [29,61].

SM is classified as indolent SM (ISM), smoldering (SSM), SM with associated hematologic neoplasm (SM-AHN), advanced SM (ASM), and mast cell leukemia (MCL) based on additional criteria described as B-findings and C-findings which depict the extent of atypical MC burden and the presence of end-organ dysfunction, respectively (Box 1 and Box 2) [61,62]. ISM is the most common with excellent survival outcomes which are comparable to that of the general population [63]. Both ISM and SSM have MC infiltration without organ dysfunction, but the presence of one or more C-findings is indicative of a more aggressive disease, either ASM (<20% MC on bone marrow smear) or MCL (≥20% MC on bone marrow smear). SM-AHN is variant of SM in which a clonal hematologic neoplasm of non-mast cell lineage is also present [55]. Bone marrow mastocytosis (BMMC), which is discussed later, may be considered a variant of indolent disease, while aggressive mastocytosis, mastocytosis with associated hematological disease, and mast cell leukemia are referred to as advanced systemic mastocytosis (Table 1).

Given that mastocytosis may affect more than one organ system, the presenting symptoms are widely variable. Mediator-related symptoms including varying degrees of pruritis, flushing, abdominal pain, nausea and vomiting, diarrhea, and bone pain are common [37,64]. Skin findings can be observed in both CM and SM, and the distribution of skin findings may range from a single lesion to more diffuse cutaneous involvement. Neuropsychiatric disturbances are often present and include symptoms of depression, anxiety, irritability, or mood swings [65]. Anaphylaxis is recognized as a life-threatening complication of mastocytosis with a reported incidence as high as 49% in one trial, and most commonly occurs in indolent SM [66]. Other symptoms associated with SM include generalized weakness, fatigue, arthralgias, myalgias, and night sweats.

Depending on the underlying type and extent of SM, osteolysis with low bone density and resultant fractures, adenopathy, splenomegaly, hypersplenism, hepatomegaly, cytopenia, and diarrhea with malabsorption syndrome may occur [19,31]. As Pardani notes, organopathy becomes more prominent as the disease progresses in severity from prediagnostic SM to mast cell leukemia [31]. Known triggers of anaphylaxis include exercise, foods, medications, or Hymenoptera stings, but in some cases unexplained anaphylaxis may be the first sign prompting further evaluation of a mast cell disorder [67]. 

It is essential to differentiate KIT gain-of-function mutation positive SM from myeloproliferative neoplasia associated with eosinophilia, splenomegaly, biochemical abnormalities (such as high tryptase and B12), and elevated bone marrow mast cell numbers [31]. In the case of the latter, somatic gene rearrangements occur (such as FIP1LI-PDGFRA or the PDGFRB gene sequence) detected by next generation sequencing (NGS) or fluorescence in situ hybridization (FISH), and such diseases are responsive to imatinib.

An activating *KIT D816V* mutation is observed in 80–90% of all cases of adult SM [55,57,62,68]. The *KIT D816V* mutation is a point mutation located on exon 17, which substitutes a valine amino acid in place of aspartate and subsequently results in constitutively active *KIT*-mediated downstream cellular signaling that influences MC differentiation [69]. Other functionally analogous *KIT* mutations have been reported affecting exons 8, 9, 10, 11, 13, and 18, although these occur rarely [70]. Mastocytosis occurring in childhood is an exception in that *KIT D816V* mutation is observed in only 40% of cases, other *KIT* mutations in 35% of cases, and wild-type *KIT* in 25% of cases [57,70].

The presence of *KIT D816V* mutation is a relatively weak contributor to malignant potential with the greatest effect on mast cell differentiation rather than mast cell proliferation [55]. In a retrospective review of 342 patients diagnosed with SM by WHO criteria, the prevalence of *KIT D816V* mutation was not significantly different across SM variants; however, patients diagnosed with indolent SM demonstrated superior survival outcomes compared with those of non-indolent SM subtypes [63]. With the introduction of NGS panels, 10 additional genes have been identified as recurrently mutated and prognostic in systemic mastocytosis, most commonly occurring in *TET2*, *SRSF2*, *ASXL1*, and *RUNX1.* Other less common mutations involve *CBL, EZH2, JAK2,* and *N/KRAS* genes [71,72,73,74,75,76].

Compound somatic mutations are most frequently observed in advanced SM variants [71,72]. In a trial of 39 patients with *KIT D816V* mutated SM, 89% of patients with ASM were found to have at least one additional non-*KIT* mutation compared with only 25% of patients diagnosed with indolent or smoldering SM. Additionally, advanced SM was also more likely to have a higher mutational burden with 78% identified with ≥3 mutations, and 41% with ≥5 mutations [71]. In a larger trial which included 150 patients with SM, the highest frequency of non-*KIT* mutations was observed in SM-AHN compared to only 14% observed in ISM patients [72]. Inferior survival is observed particularly among patients harboring one or more mutations affecting the *SRSF2, ASXL1,* and *RUNX1* genes or in patients with complex karyotypes [73,74]. In a trial of 70 patients, mutation in *SRFSF2* or *ASXL1* were independent risk factors for worsened overall survival on multivariate analysis, and survival outcomes were further influenced by the number of mutated genes on the *SRFSF2/ASXL1/RUNX1* (S/A/R) gene panel [73]. In addition, S/A/R^pos^ is associated with poor response rates to targeted pharmacotherapy in advanced SM disease [77]. Among patients without any detectable mutations of the S/A/R panel, the presence of *EZH2* mutation identified a group of patients in one study with worsened progression-free and overall survival. Thus, the addition of *EZH2* status to the S/A/R panel may have greater predictive value for survival outcomes compared with the S/A/R panel alone [75]. *TET2* has also been implicated as a poor prognostic factor [76]. 

## 9. Nonclonal Disorder: Mast Cell Sarcoma

Patients with mast cell sarcoma (MCS) typically present with a localized destructive growth composed of mast cells but lacking bone marrow changes or other biomarkers of SM [31,78,79]. High-grade cytology is typical, and most mast cells appear atypical in morphology. Monnier and coworkers described 23 cases of MCS and commented on the difficulty in diagnosis due to the atypical appearance of the cells and the resultant confusion that arises in differentiating this condition from other malignancies [79]. The prognosis is poor with less than two years survival from the time of diagnosis. *KIT 816V* mutation occurs in one-fifth of the patients evaluated, but the disease is resistant to most conventional therapies [79]. Symptoms of mast cell activation syndrome can occur in one-third of patients with MCS but, the majority present with mass lesions that can occur in any organ in the body: bones, the liver, the spleen, or lymph nodes [79].

## 10. Mast Cell Activation Syndrome 

**Figure 4:**
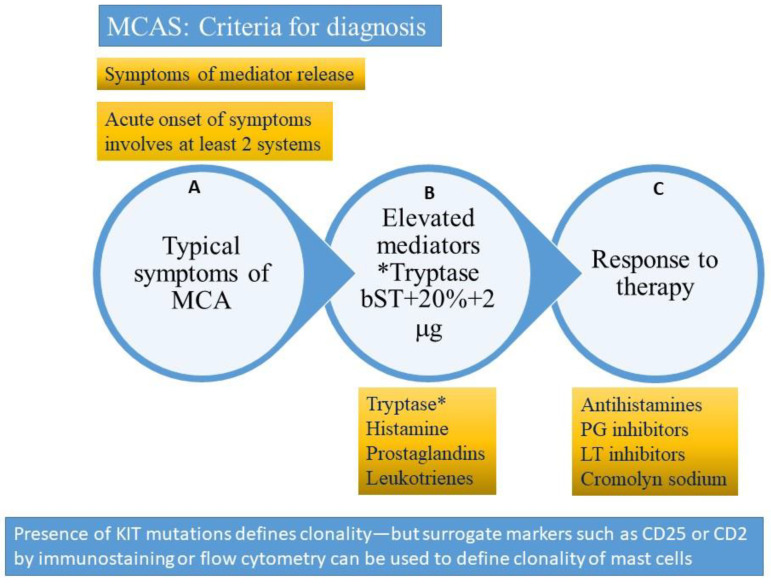
Criteria for diagnosis of mast cell activating syndrome (please refer to text for description). A = clinical criterion, B = laboratory criterion, C = treatment criterion. All three criteria need to be satisfied to confirm a diagnosis of mast cell activation syndrome (MCAS). The presence of clonality (*KIT D816V* mutation or surface expression of CD2 or CD25 clonal markers) would suggest a diagnosis of clonal (primary) mast cell activating syndrome also referred to as the monoclonal mast cell activation syndrome (MMAS). * Tryptase (total tryptase) is usually used as a surrogate marker for mast cell activation. PG = prostaglandin, LT-leukotriene, MCA = mast cell activation. bST = basal serum tryptase.

Mast cell activation syndromes (MCAS) are a clustering of disorders characterized by the appearance of symptoms related to mast cell degranulation and mediator release. The typical syndrome is characterized by (1) pathological accumulation of mast cells in organs and tissues and/or (2) aberrant release of ≥mast cell mediators. MCAS are classified as primary clonal (clonal disorders which include systemic mastocytosis and its variants, cutaneous mastocytosis, and the clonal mast cell activating syndrome (CMCAS) or monoclonal mast cell activating syndrome (MMAS)) and the non-clonal mast cell activating syndromes, including secondary, idiopathic, and combined disorders (Table 1) [27,28,52,80,81]. Patients with mastocytosis meet the World Health Organization (WHO) criteria for either cutaneous or systemic disease [31]. The criteria for mast cell activating syndrome were first established in 2012 and then refined in 2019 [28]. These are reviewed below and detailed in Figure 4 and Box 3.

Box 3 **Criteria suggested for the Diagnosis of Mast Cell Activating Syndrome and its Known Variants.**

Mast Cell Activation Syndromes (MCAS)

A condition characterized by episodic symptoms arising from mast cell mediator release and needs to meet 3 criteria:

1. Acute and recurrent symptoms arising from mast cell activation and degranulation with at least 2 organ systems affected at presentation (skin, gastrointestinal, respiratory, neuropsychiatric or cardiovascular)
2. Elevated mast cell-derived mediators (usually tryptase increase using formula 20% + 2 ng/mL over basal tryptase) during episode
3. Improvement of symptoms after treatment with inhibitors of mast cell mediator release or function

Primary Clonal (or Monoclonal) Mast Cell Activation Syndrome (CMCAS/MMAS)

1. Does not meet criteria for systemic mastocytosis
2. Meets criteria for MCAS as above
3. Clonality is present
  - KIT Asp816Val mutation in >80% cases (in tissue, peripheral blood or might require bone marrow evaluation) by next generation sequencing or allele-specific polymerase chain reaction
  - Otherrarer mutations (*TET2, SRSF2, ASXL1, RUNX1, CBL, JAK2*) are observed more in systemic mastocytosis than MMAS
  - Expressionof CD2 (lymphocyte function-associated antigen 2) and/or CD25 (alpha chain ofhigh affinity receptor for interleukin 2) markers on mast cells by immunostaining or by flow cytometry

Secondary Mast Cell Activation Syndrome

1. Does not meet criteria for systemic mastocytosis
2. Meets criteria for MCAS
3. Mast cell clonality is absent
4. Underlying etiology for mast cell activation is usually present (allergy to medications or foods, autoimmunity, neoplasia or infection)

Idiopathic Mast Cell Activation Syndrome

1. Does not meet criteria for systemic mastocytosis
2. Meets criteria for MCAS
3. Mast cell clonality is absent
4. Underlying trigger for mast cell activation is usually absent

Combined Disorder

  
Combination of clonal mast cell activation (CMCAS) and underlying allergy

## 11. Diagnosis of Mast Cell Activation Syndrome 

The diagnosis of MCAS can be made if specific criteria are met (Figure 4), namely the following: 

1. Firstly, the diagnosis can be made if signs and symptoms of mast cell activation are present involving at least two organ systems [27,28,52,80,81,82,83]. The symptoms need to be acute in onset and characterized by recurrent episodes (some requiring emergency room visits or hospitalizations allowing documentation). Symptoms are like those described with anaphylaxis, and include mucocutaneous (flushing, urticaria, pruritus, angioedema), gastrointestinal (diarrhea, nausea, vomiting, crampy abdominal pain), respiratory (wheezing, dyspnea, inspiratory stridor), cardiac (hypotension, tachycardia), and neurological involvement (pre-syncope, syncope) [27,28,52,80,81,82,83]. The differential diagnosis is extensive and should include toxic, endocrine, infectious, vascular, metabolic, inflammatory, and neoplastic conditions [28,82]. Care must be taken to avoid misdiagnosing hereditary angioedema, acquired angioedema, hyper-eosinophilic syndromes, neuroendocrine tumors (gastrinoma, VIPoma, medullary thyroid carcinoma, pheochromocytoma, and carcinoid syndrome), adrenal disease, aspirin-exacerbated respiratory disease, scombroid poisoning, inflammatory bowel disease, hypothyroidism, and renal insufficiency. Obtaining a detailed history, performing a thorough physical examination, and evaluating specific laboratory abnormalities (levels of tryptase, cortisol, N-methylhistamine, 5-hydroxy indoleacetic acid, metanephrines, thyroid stimulating hormone, cortisol, gastrin, VIP, calcitonin, and measuring renal function) can help in differentiating these conditions [28]. 

2. Secondly, the diagnosis can be made with laboratory confirmation of mast cell mediators as measured by elevation of tryptase, histamine, or its metabolites. Tryptase is often the mediator used to monitor mast cell degranulation. It is a mast cell specific mediator that needs to be measured within 4 h of mast cell activation or the onset of anaphylactic symptoms [27,28]. Tryptase circulates as α-Pro-tryptase in the serum and is the dominant detectable form in patients with SM [8]. On the other hand, the dominant form circulating during anaphylaxis is β-tryptase. This form of tryptase peaks 30–90 min after mast cell degranulation with a half-life of 4 h [8]. Most commercial assays measure total tryptase (which is a sum of the α and β components), which is a surrogate measure of mast cell burden, with the medium level in healthy individuals averaging about 5 ng/mL and with most individuals having levels less than 15 ng/mL.

Tryptase levels obtained after an acute episode should be compared with baseline tryptase levels (sBT). This could be a recent tryptase drawn prior to the event or within 48 h after the event. The formula for a significant increase in tryptase level is sBT × 1.2 + 2 ng/mL; for example, if basal tryptase level is 10 ng/mL, a level within 4 h of an anaphylactic episode of 14 ng/mL would be considered significant [28,39,84]. Two or more such elevations would be considered diagnostic following acute, recurrent episodes [80]. Tryptase levels may be elevated in many conditions, including hyper-eosinophilic syndromes, hematological malignancies, myelodysplastic syndrome, atopic disease, helminth infestation, end-stage kidney disease, hereditary alpha tryptasemia, and on occasion, normal variants or false positivity related to heterophilic antibodies interfering with laboratory assays [16]. These conditions need to be carefully evaluated for and excluded before a diagnosis of SM or MCAS is made.

Other laboratory tests that may be considered in lieu of tryptase include urinary N-methyl histamine, 9α-11β-prostaglandin F2 (11β-PGF2α), and leukotriene E_4_ level [8,85]. In one study by Ravi et al., a combination of serum tryptase and 11β-PGF2α helped the avoid misdiagnosis of MCAS in a group of 25 patients [85]. The authors felt such patients may also benefit from prostaglandin inhibition using salicylates. 11β-PGF2α appears to be a better marker than tryptase for MCAS, but assays are difficult to perform and only available in a few limited laboratories. Moreover, unlike tryptase, the other tests have not been standardized or compared in well conducted studies. While earlier tests required a 24-h urine collection, more recent measurements are done on spot urine specimens collected after the patient empties his or her bladder during an acute event [28]. Levels greater than 30% above the upper limits of normal can be considered abnormal [28].

3. Thirdly, the diagnosis can be made if there is a favorable response to agents which act as mast cell stabilizers or inhibitors of mast cell mediators. These are reviewed later, but include histamine receptor antagonists, leukotriene blockers, mast cell stabilizers, and aspirin or nonsteroidal anti-inflammatory agents [28,81,84]. Selected cases will require immune suppression or cytoreductive therapy as well, especially for patients suffering from aggressive clonal disease. 

## 12. Primary or Clonal Mast Cell Activation Syndrome (MMAS or CMCAS)

Clonal MCAS (also referred to as MMAS) is characterized by mast cell expression of clonal markers, either c-KIT mutation (D816V and rarely other mutations) and/or aberrant expression of CD25 or CD2 markers on the surface of mast cells.

In the primary disorders, the mast cells are abnormal and in a hyperactive state and degranulate spontaneously or in response to other triggers. The primary conditions include cutaneous mastocytosis (diagnostic criteria for CM are met but not for SM), systemic mastocytosis (meets criteria for SM: one major and one minor or three minor criteria) and clonal MCAS (when only two minor SM criteria are met, such as clonality and high tryptase or an abnormal shape of mast cells).

The most relevant finding to classify the MCAS is the presence versus absence of clonal MCs as defined by the presence of c-KIT mutations and/or aberrant expression of CD25 and/or CD2 on mast cells. However, other criteria that confirm systemic mastocytosis, listed in Box 1 (especially mast cell aggregates in bone marrow, presence of cutaneous disease, and tryptase levels > 20 ng/mL) are not met [27,28,52,80,81]. Thus, patients with monoclonal mast cell activation syndrome (MMAS) typically demonstrate monoclonal mast cell populations but without abnormal proliferation or aggregation. These patients also lack typical skin lesions and their tryptase levels are usually less than 20 ng/mL. The Spanish Network on Mastocytosis Score (REMA) helps predict patients who might have an underlying clonal disorder and require subsequent bone marrow biopsy. 

## 13. Secondary (Non-Clonal) Mast Cell Activating Syndrome 

Other mast cell activating conditions include idiopathic MCAS or non-clonal or secondary MCAS (NC-MCAS). Patients with idiopathic MCAS (discussed in more detail in the next section) present with signs and symptoms of mast cell activation but in the absence of primary and secondary causes or hereditary alpha tryptasemia (HαT). Others have labeled these subjects as having non-clonal mast cell activation syndrome (NC-MAS). In secondary MCAS, the mast cells are normal but are activated by allergens and other physical or chemical stimuli. These conditions include IgE-mediated allergic/atopic diseases, drug reactions, mast cell hyperplasia secondary to autoimmune diseases such as autoimmune urticaria, and chronic infections, and chronic inflammatory diseases such as rheumatoid arthritis or inflammatory bowel disease.

## 14. Combined MCAS 

Sometimes a primary mast cell activation (clonal) disorder may be complicated by secondary IgE-mediated hypersensitivity mast cell reactions to food, medications, or insect stings.

## 15. Idiopathic Anaphylaxis and Idiopathic Mast Cell Activation 

Idiopathic anaphylaxis (IA) is a condition characterized by repeated episodes of anaphylaxis with no demonstrable external trigger [86]. Human MCs as well as basophils may be responsible for the manifestation of anaphylaxis that accompanies the condition. IA was described about four decades ago and since then the classification and management of the disease have been defined. Typically, there is involvement of two or more systems (such as cardiovascular, respiratory, or gastrointestinal) and patients often present to the emergency room or urgent care with urticaria, angioedema of the lips or tongue, wheezing, vomiting, diarrhea, and confusion or syncope [86]. Fatality due to IA is rare but has been recorded. Thirty to sixty percent of adults and 10% of children with anaphylaxis may have no demonstrable etiology and are hence classified as IA, with a predilection for women and patients with underlying atopic disease. More recently, exclusion of alpha-gal allergy (delayed allergic reactions to red meat), and primary mast cell and somatoform disorders are required before the diagnosis is made [5,82,83,87]. Grammar and coworkers showed that, compared to patients with mast cell disorders, IA patients have evidence of activated T cells and B cells in the peripheral blood, although it is unclear whether this is primary or an epiphenomenon, as some of the patients with activated CD19+ CD23+ B cells were also receiving concomitant glucocorticoids [88]. One unpublished study that used gene expression profiling showed that patients with IA had activated genes regulating mast cell and basophil degranulation [86]. In many situations, IA may be difficult to differentiate from idiopathic mast cell activation syndrome. In the absence of a clear-cut diagnosis of anaphylaxis, alternative etiologies need to be considered, including vocal cord dysfunction, flushing disorders (carcinoid syndrome, pheochromocytoma, VIP secreting tumors), vasovagal syncope, panic attacks, scombroid poisoning, and a variety of cardiopulmonary disorders [87]. In general, young males with hypotension or syncope in the absence of urticaria or angioedema are more likely to have a clonal neoplasm, in contrast to IA, where most patients presented with urticaria and/or angioedema [89]. 

## 16. Hymenoptera Reactions and Mast Cell Disorders

Hymenoptera venom allergy can result in serious, and potentially fatal, systemic reactions [90,91]. Patients with SM and MCADs are known to have a higher risk of severe reaction to insect stings, and, thus identifying systemic mast cell disease in patients with a Hymenoptera venom allergy has serious clinical implications due to the increased risk of life-threatening reactions in this patient population. The concomitant use of beta-blockade and/or angiotensin converting enzyme inhibitor therapies poses additional risk. Recent studies suggest that anaphylaxis to Hymenoptera venom (especially hypotension or syncope) can indicate the presence of an underlying clonal mast cell disorder [91]. Selb et al. recommend routine *KIT D816V* gene mutation screening in these patients, and that KIT mutation may be a more sensitive biomarker than serum tryptase levels [92]. Of 351 patients with normal serum tryptase levels, they observed *KIT D816V* gene mutation by peripheral blood analysis in 28 patients, predominantly occurring in those patients with a history of more severe anaphylactic reactions [92]. Among patients with normal tryptase levels, KIT mutation was regarded as the major risk factor for severe Hymenoptera reactions. In patients with elevated tryptase, hereditary alpha tryptasemia (HαT) along with KIT mutation contributed to >90% of basal serum tryptase (sBT) elevations in patients with a severe Hymenoptera allergy.

According to Bonadonna and colleagues, Hymenoptera venom allergy is the most common allergic reaction in patients with primary clonal mast cell activation syndrome and thus remains a major area of clinical investigation [6]. With estimates as high as 7.9%, the prevalence of Hymenoptera venom allergy associated with systemic mast cell disease exceeds the observed prevalence in the general population [93]. Interestingly, and for unknown reasons, venom allergy is absent in patients with aggressive systemic mastocytosis and increased mast cell load [6].

The typical demographic of patients with primary mast cell disorders and concomitant Hymenoptera venom allergy tends to be male with a unique predominance of anaphylactic shock and lower basal serum tryptase elevations, but without cutaneous findings [91,93]. As normal basal serum tryptase alone is not sufficient to exclude a diagnosis of mastocytosis; it is important to consider bone marrow biopsy in patients with severe venom reactions associated with hypotension and syncope in the absence of cutaneous findings and regardless of serum tryptase level [91]. According to Vos et al., 97.5% of patients with systemic mast cell disease experience subsequent systemic reactions, where 90% of these reactions are severe in nature in the absence of immunotherapy treatment [90].

Asymptomatic sensitization to Hymenoptera venom is common and requires a history of prior reaction to a sting to allow testing. All patients presenting with a Hymenoptera venom allergy are recommended to undergo screening with a basal serum tryptase level to identify co-existing mast cell disease [93,94,95]. Additional diagnostic work-up for Hymenoptera venom allergy consists of lab testing for serum IgE directed to the specific allergen and/or skin prick test to the various venoms [6,96]. Identifying co-existing Hymenoptera venom allergy in mast cell disease has historically been clinically challenging due to the low sensitivity of venom specific IgE levels [6]; however, with a lower threshold for venom specific IgE levels (IgE > 0.17 kU_A_/L), the diagnostic sensitivity has been improved [90,95].

Life-long venom immunotherapy is the mainstay of treatment for patients with systemic mastocytosis and Hymenoptera venom allergy [91,94,95]. Concerns regarding the therapeutic safety of venom specific immunotherapy has led to debate of whether it should be routinely used as these patients are more prone to experiencing severe adverse reactions to therapy. Nonetheless, previous studies have suggested that the benefits of lifelong treatment outweigh the potential risks [91,95].

## 17. Bone Marrow Mastocytosis

Bone marrow mastocytosis (BMMC) is a variant of ISM presenting with clonal mast cell proliferation of the bone marrow without any evidence of additional extracutaneous mast cell infiltration. Anaphylaxis is more frequently observed in BMMC with an estimated incidence of >90%, compared to 20–49% in patients with ISM, and 0–2% in the general population [97]. Patients usually lack skin manifestations, and bone marrow may sometimes demonstrate little to absent aggregate formation; therefore, diagnosis is difficult and requires a high index of suspicion on the part of both the physician and the pathologist [98]. A gross pathology review of bone marrow specimens may be unremarkable and require immunostaining to demonstrate abnormal mast cells [98]. Accordingly, four categories of bone marrow findings in patients with mast cell disorders have been described: (1) classical aggregates (as seen in SM); (2) abnormal, dispersed mast cells without aggregates but with unusual morphology or surface expression of clonal markers (CD25 or CD2) as seen in BMMC; (3) subclinical bone marrow with findings consistent with a mast cell activation syndrome (MCAS); and (4) normal bone marrow examination [98]. The clinical course is indolent, but some patients may present with Hymenoptera anaphylaxis or cryptic osteoporosis [97].

## 18. Hereditary Alpha Tryptasemia

Hereditary α-tryptasemia (HαT) is the most common cause of elevated baseline tryptase. A defining feature is elevated serum basal tryptase > 8 ng/mL due to increased pro-alpha-tryptase synthesis rather than increased mast cell activation [99]. It is inherited in an autosomal dominant pattern with an estimated incidence of 5% in the general population. Human tryptase is encoded by genes located on chromosome 16, and three regulatory genes have been described which effect alpha (α), beta (β), and gamma (γ) tryptase expression. Of these, TPSB2 encodes β-tryptase and TPSAB1 encodes either α- or β-tryptase [51]. The genes TPSABI and TPSB2 can be assessed by the digital drop PCR technique, which usually involves studying a buccal cell sample, and duplications, triplications, and quintuplications are associated with corresponding elevations in basal tryptase levels in the serum.

One of the mechanisms involved in disease pathogenesis is the formation of α/β tryptase tetramers, which have been shown to cleave and activate two receptors: EMR2 (EGF-like module-containing mucin-like hormone receptor-like 2, also known as CD312, is a protein encoded by the ADGRE2 gene, a member of the adhesion G protein-coupled receptor family) and PAR2 (protease activated receptor 2, also known as coagulation factor II (thrombin) receptor-like 1 (F2RL1) or G-protein coupled receptor 11 (GPR11), is a protein that in humans is encoded by the F2RL1 gene) [100]. Activation of these receptors may be involved in increased vascular leakage and edema formation, which subsequently leads to the manifestations of urticaria, angioedema, and anaphylaxis [100]. Mature tryptases have also been shown to activate anaphylatoxins and the contact system, which may have additional contributions to anaphylaxis (such as inflammatory/chemotactic responses and coagulation abnormalities) [5,101,102,103]. Complement can activate mast cells to express chymase and tryptase (via complement receptors), which in turn can further activate complement, leading to a fascinating paracrine mechanism of mast cell activation in inflammation [103].

HαT is known to contribute to severe anaphylactic reactions in patients with venom allergy and can also modify the severity of symptoms arising from most mast cell disorders, including SM and MCADs [100]. The prevalence of HαT is increased in both clonal and non-clonal disease and hence an assessment for this condition may be of importance in the evaluation of mast cell disorders [100]. Patients have elevated baseline serum tryptase level and multiple copies of TPSAB1 gene encoding α-tryptase. Elevated tryptase levels >11.4 ng/mL are common in the population (4–6%), and besides HαT, the underlying reason may be renal failure, infection, myeloproliferative neoplasm, or mast cell disorders [100]. Patients present with a variety of symptoms including flushing, irritable bowel syndrome, or symptoms of urticaria, angioedema, and/or anaphylaxis. In one study of 101 patients with HαT, 80% were female, with an average tryptase level of 17.2 ng/mL, and over half presented with unprovoked anaphylaxis but many presented with neurological, pulmonary, cardiovascular, gastrointestinal, cutaneous, and psychiatric disturbances [104]. While antihistamines helped control symptoms, omalizumab was effective in decreasing urticaria and anaphylaxis in over 90% of the patients [104]. In general, it is expected that one-third of patients will develop severe symptoms, one-third show moderate symptoms, and one-third are often asymptomatic [99]. Asthma, rhinitis, urticaria, pruritus, abdominal pain, and food allergy respond to omalizumab therapy while dysautonomia, joint hypermobility, and diarrhea are more refractory to therapy [99].

## 19. Combined Disorders

Various combinations of primary clonal mast cell disorders (ISM, SSM, ASM, MCAS), secondary mast cell activation (triggers including drugs, venom, latex, foods), and HαT have been described, especially since atopic disease (20–30% of the population) and HαT (6% of the population) can be considered as relatively common conditions.

## 20. Management of Mast Cell Disorders

A detailed discussion of the management of these conditions is beyond the scope of this review and the reader is referred to excellent reviews on the topic listed below. The principles of disease management of non-advanced variants of mastocytosis are focused primarily on trigger avoidance and symptom control, and inhibition of pharmacologic mediators can be individualized to specific needs (Table 2). In cutaneous disease, topical corticosteroids are an effective option for lesions affecting a limited surface of distribution, and oral corticosteroids can be utilized in more severe cases. In steroid-refractory mastocytosis of the skin, ultraviolent phototherapy in combination with or without oral psoralen can be an effective alternative treatment option [36]. The use of other novel medications such as omalizumab as an intervention has been recommended on a case-by-case basis [105,106,107,108,109,110,111,112,113].

Antihistamine receptor antagonists are often chosen in the front-line setting for the management of mediator-related symptoms (H_1_-receptor antagonists), namely pruritis and flushing, or for gastrointestinal symptoms (H_2_-receptor antagonists) including abdominal pain, cramping, dyspepsia, or diarrhea [61,67,82,114,115]. Proton pump inhibitors (PPI) and/or cromolyn sodium may be added as adjunctive therapy for break-through gastrointestinal symptoms that are not responsive to H_2_-antagonists. Adjunct therapy with leukotriene receptor antagonists is effective for refractory mediator-mediated symptoms [31,67]. Aspirin may be considered in more severe cases, although treatment should be approached cautiously due to the potential for mast cell activation and gastrointestinal toxicities associated with long-term use [28,67,116].

Given the high incidence of anaphylaxis, patients with mastocytosis should carry self-injectable epinephrine for emergency use as needed. In addition, short-acting H_1_-receptor antagonists, such as diphenhydramine or hydroxyzine, and oral corticosteroids should be used for sub-acute symptom management following an anaphylactic episode [28,67,82,83,92]. An epinephrine autoinjector should always be prescribed for patients presenting with anaphylaxis or severe systemic reactions including syncope [82,83]. For patients with recurrent episodes of anaphylaxis, the anti-IgE monoclonal antibody omalizumab has been shown to significantly improve symptom control and improve quality of life [105,106,107,108,109,110,111,112,113]. Other biologicals that target mast cell regulatory proteins and receptors—KIT, IgE, SIGLEC-8, IL-4 receptors, IL-6 receptors, IL-9, IL-33, tryptase, CD25, and CD30— are in development or undergoing clinical trials [117].

Targeted therapeutic agents with disease-modifying activity are reserved for progressive and/or aggressive SM (Table 3). As *KIT D816V* mutation is present in most cases, it has become a primary target for systemic treatment. Midostaurin and the recently FDA-approved avapritinib are two tyrosine kinase inhibitors (TKI) which have activity in *KIT D816V*-mutated disease [118,119,120]. In patients with ASM, midostaurin demonstrated an overall response rate (ORR) of 60%, including 45% with an observed major response defined as complete resolution of at least one disease-related organ dysfunction. The response rates were similar across sub-types, including in 16 patients with mast cell leukemia (MCL) [121]. The selective *KIT D816V* inhibitor avapritinib was recently FDA-approved for the treatment of ASM in June 2021 based on the emerging results of the phase I EXPLORER trial and phase II PATHFINDER trial [122,123]. At an interim analysis of the phase II PATHFINDER trial, ORR was 75%, and there was no observed difference in response rates between previously treated and untreated patients, including those receiving prior midostaurin therapy [122].

Alternative TKI therapies are available, although they have a limited role in the current treatment paradigm of SM. Imatinib and Masitinib have shown activity in SM, but both agents are inactive against *KIT D816V* [68,70]. Nilotinib and dasatinib have limited efficacy with reported response rates of 22% and 33%, respectively [124,125]. The role of hematopoietic stem cell transplant (HSCT) is less well defined in systemic mastocytosis, and to date there are no prospective trials to guide which groups of patients derive the greatest benefit, the optimal timing of transplantation, or the preferred conditioning regimens [68].

## 21. Unmet Needs and Areas of Uncertainty

The roles of clonality and novel mutations of KIT or other genes involved in mast cell activation are unknown. The role of biological agents such as omalizumab, dupilumab, or mepolizumab are unknown or reported in anecdotal cases without solid randomized studies. Other than tryptase, it is unclear as to which mediators can be measured readily and reproducibly in emergency rooms and intensive care units. Even tryptase, though a well-known and readily available test, is not always ordered during an episode. There are other vague conditions such as joint hypermobility syndromes and postural orthostatic tachycardia where a role for mast cell activation is invoked without much concrete data [27]. The interactions between HαT, clonal and non-clonal mastocytosis, and the observed overlap with idiopathic anaphylaxis need further definition and clarification. The role for more aggressive bone marrow evaluation when tryptase levels are well below 20 ng/mL (threshold for bone marrow evaluation) needs to be defined and explored.

## 22. Conclusions

Mastocytosis, mast cell activation disorders, idiopathic anaphylaxis, and hereditary alpha tryptasemia are conditions encountered in the evaluation of patients with recurrent severe allergic reactions. Initially a cluster of vague and poorly defined diseases, these diseases are now readily diagnosed with specific laboratory tests, using biomarkers such as tryptase and/or skin, bone marrow, or tissue examination. New therapies are available, and the management of these disorders has also been influenced by the development of biologicals and specific immune inhibitors.

## Figures and Tables

**Table 1:** Classification of Disorders Associated with Mast Cells.

**1.** **MASTOCYTOSIS** **a.** Cutaneous Mastocytosis * Diffuse cutaneous mastocytosis Maculopapular mastocytosis (Urticaria Pigmentosa) Cutaneous mastocytoma **b.** Systemic Mastocytosis * Indolent systemic mastocytosis (ISM) [includes bone marrow mastocytosis] Smoldering systemic mastocytosis (SSM) **c.** Advanced Systemic Mastocytosis * Aggressive systemic mastocytosis (ASM) Mastocytosis with associated hematological neoplasia (SM-AHN) Mast cell leukemia (including aleukemic leukemia) **d.** **Nonclonal disorders: Mast cell sarcoma** **2.** **MAST CELL ACTIVATION SYNDROME (MCAS)** **a.** Primary Mast cell Activating Syndrome/Monoclonal Mast Cell Activating Syndrome (MMAS)/Clonal Mast Cell Activating Syndrome (CMCAS) * **b.** **Secondary Mast Cell Activating Syndrome** (IgE-mediated allergy, autoim-munity, chronic infection, nematode infestation, underlying neoplasia) **c.** **Idiopathic Mast Cell Activation Syndrome** (no obvious etiology) **d.** **Combined primary disorder with allergy-triggered mast cell activation** **3.** **IDIOPATHIC ANAPHYLAXIS (IA)** No obvious allergens (cryptic allergens and mammalian meat allergy to be excluded) Idiopathic anaphylaxis associated with bone marrow mastocytosis and clon-ality * IA associated with CMCAS and/or hereditary alpha-tryptasemia * **4.** **REACTIONS TO HYMENOPTERA VENOM AND CLONAL MAST CELL DIS-EASE** Associated with bone marrow mastocytosis and clonality * Associated with MCAS and/or clonality * **5.** **BONE MARROW MASTOCYTOSIS** **6.** **HEREDITARY ALPHA TRYPTASEMIA** Isolated condition Associated with CMCAS, SM and variants and IA **7.** **COMBINED DISORDERS** (Various combination of above conditions)

* = Associated with clonality (D816V mutation, other mutations or associated with aberrant mast cell expression of CD2 or CD25 on cell surface).

**Table 2:** Medical Management of Mast Cell Disorders.

Drug Class	Medication and Dosing	Primary Indication and Comments
**H_1_-receptor** **antagonist**	*Long-acting*: Fexofenadine (60–180 mg orally once or twice/day) Cetrizine (5–10 mg orally once or twice/daily) Loratidine (10 mg orally once daily) *Short-acting*: Diphenhydramine (25–50 mg orally or parenterally every 6-8-h as needed) Hydroxyzine (25 mg orally every 6-h as needed)	Histamine-related symptoms (i.e., flushing, pruritis)
**H_2_-receptor** **antagonist**	Ranitidine (150 mg orally, twice/day) Famotidine (10 mg orally twice/day)	Gastrointestinal symptoms
**Proton-Pump** **Inhibitor (PPI)**	Omeprazole (20–40 mg orally daily Pantoprazole (20–40 mg orally daily)	Gastrointestinal symptoms
**Leukotriene** **receptor antagonist**	Montelukast (10 mg orally daily Zafirlukast (20 mg orally daily)	Histamine-related symptoms (i.e., flushing, pruritis)
**Mast cell stabilizer**	Cromolyn sodium (four times a day maximum daily dose 40 mg/kg/day)	Gastrointestinal symptoms Histamine-related symptoms (i.e., flushing, pruritis)
**Non-steroidal anti-inflammatory drug (NSAID)**	Aspirin (variable dosing)	Histamine-related symptoms (i.e., flushing, pruritis)
**Anti-IgE** **monoclonal** **antibody**	Omalizumab (150–300 mg administered subcutaneously every 2–4 weeks)	Histamine-related symptoms (i.e., flushing, pruritis, recurrent anaphylaxis) not responsive to conservative measures
**Immediate administration for anaphylaxis**	Epinephrine (0.3–0.5 mg intramuscularly every 5–15 min in the lateral thigh)	Anaphylaxis
**Cytoreductive therapy**	Interferon-α (IFN-α) (Starting dose of 3 million units subcutaneously three times weekly can be increased to 3–5 million units up to 5 times weekly) Cladribine (2-CdA) (5 mg/m^2^/day or 0.14 mg/kg/day as a 2-h infusion for 5 days. Up to 6 cycles every 4–8 weeks)	IFN-α: often co-administer with prednisone to improve tolerability Flu-like syndrome 2-CdA: myelosuppression and lymphopenia common. Prophylaxis for Pneumocystis jirovecii infection needed

Abbreviations: mg, milligrams; PO, per oral; BID, twice daily; PRN, as needed; QID, four times daily; kg, kilograms; SQ, subcutaneous; IM, intramuscular; MU, million units.

**Table 3:** Tyrosine kinase inhibitors used in Mast Cell Disorders.

Drug	Drug Target	Dosing	Comments
Midostaurin *	Multi-kinase inhibitor of *KIT*, *VEGFR*, *FLT3*, *PDGFR*	100 mg PO BID	Multi-kinase inhibitor with activity against *KIT-D816V*
Avapritinib *	Selective *KIT-D816V* inhibitor	200 mg PO daily	Selective *KIT-D816V* inhibition; Effective in patients having received prior Midostaurin therapy
**Imatinib**	Multi-kinase inhibitor of *KIT*, *PDGFR*, *Bcr-Abl*	400 mg PO daily	Resistance observed in *KIT-D816V*
**Masitinib**	Multi-kinase inhibitor of *KIT*, *PDGFR*, *Lyn*, *FGFR3*	6 mg/kg PO divided into two daily doses	Resistance observed in *KIT-D816V*
**Dasatinib**	Multi-kinase inhibitor of *KIT*, *PDGFR*, *Bcr-Abl*, *Src*	140 mg PO daily	Limited therapeutic activity in SM
**Nilotinib**	Multi-kinase inhibitor of *KIT*, *PDGFR*, *Bcr-Abl*	400 mg PO BID	Limited therapeutic activity in SM

Abbreviations: PO, per oral; BID, twice daily; mg, milligrams; kg, kilograms; SM, Systemic Mastocytosis. * FDA approval for treatment of Systemic Mastocytosis.

## Data Availability

Not applicable.
